# The Emergence and Pathogenesis of Recombinant Viruses Associated with NADC34-like Strains and the Predominant Circulating Strains of Porcine Reproductive and Respiratory Syndrome Virus in Southern China

**DOI:** 10.3390/v14081695

**Published:** 2022-07-31

**Authors:** Xindong Wang, Kang Zhang, Qingrong Mo, Guochang Chen, Jing Lv, Jing Huang, Yanli Pang, Hao Wang, Wenbo Liu, Kai Huang, Xiangling Min, Tongwei Ren, Kang Ouyang, Ying Chen, Weijian Huang, Zuzhang Wei

**Affiliations:** College of Animal Science and Technology, Guangxi University, No.100 Daxue Road, Nanning 530005, China; wangxd1015@163.com (X.W.); 15277156489@163.com (K.Z.); mqr18269003560@163.com (Q.M.); 18878876394@163.com (G.C.); lj13882924031@163.com (J.L.); hj2018302010@163.com (J.H.); 18897875366@163.com (Y.P.); wangh402001@163.com (H.W.); liuwenbo07312022@163.com (W.L.); huangkai9608@163.com (K.H.); mxl17715757956@163.com (X.M.); r17372768685@163.com (T.R.); ouyangkang@gxu.edu.cn (K.O.); yingchen@gxu.edu.cn (Y.C.); huangweijian-1@163.com (W.H.)

**Keywords:** porcine reproductive and respiratory syndrome virus, NADC34-like, genetic evolution, recombination, pathogenicity

## Abstract

Since its recent appearance in China, the NADC30-like strains of porcine reproductive and respiratory syndrome virus 2 (PRRSV-2) have caused an expanding epidemic, and this has further expanded the genetic diversity of PRRSV. In this study, three NADC30-like strains—GXFCG20210401, GXQZ20210403 and GXNN20210506—were isolated from pig serum samples obtained in Guangxi, and their genomes were sequenced. A comparative analysis of the whole genomes showed that the three strains were most similar to NADC30 (88.3–88.7%). In particular, the non-structural protein coding regions (nsp1, nsp4-5, nsp7-8 and nsp9) showed the highest similarities to JXA1, and the ORF2a-ORF5 regions showed the highest similarities to NADC34. The three strains had same discontinuous deletions of 111+1+19 amino acids in the nsp2 region, which were similar to the NADC30-like strains. Phylogenetic tree analysis based on the ORF5 gene showed that the three PRRSV isolates were divided into lineage 1.5 along with the representative NADC34-like strains, but they were classified as NADC30-like strains with respect to the whole genome and nsp2 evolutionary trees. Recombinant analysis revealed complex recombination patterns in the genomes of the three strains, which likely originated from multiple recombination events among JXA1-like, NADC30-like and NADC34-like strains. The results from animal experiments showed that the GXQZ20210403 strain was 20% lethal to piglets and caused more severe clinical reactions than GXFCG20210401, and both recombinant strains were similar in terms of pathogenicity to the previously reported NADC34 strains. This study demonstrates that NADC34-like strains of PRRSV have been circulating in the southern provinces of China and have exchanged genomes with several other indigenous strains. In addition, differences in recombination patterns may cause different clinical pathogenicity and indicate the importance of the surveillance and preventive control of recombinant strains.

## 1. Introduction

Porcine reproductive and respiratory syndrome virus (PRRSV) is an economically important swine viral pathogen which causes reproductive and respiratory disease in pigs throughout the world. PRRSV belongs to the genus Porartevirus and the family Arteriviridae [1]. PRRSVs are single-stranded, enveloped and positive-sense RNA viruses. The genome length of PRRSV is approximately 15.3 kb, which contains at least ten open reading frames (ORFs), a 5′ cap structure and a 3′ poly (A) tail [2]. The two large replicase polyproteins (pp1a and pp1ab) encoded by ORF1a and ORF1b, respectively, are proteolytically processed by virus-encoded proteases to at least 16 mature non-structural proteins (nsps) [3,4]. The ORF2-7 encodes through seven viral structural proteins including glycoprotein (GP) 2a, E, GP3, GP4, ORF5a protein, GP5, matrix protein (M) and nucleocapsid protein (N), respectively [3,5,6]. 

According to the genetic diversity, PRRSVs are mainly classified into two genotypes: type 1 (European) PRRSV and type 2 (North American) PRRSV [7]. The two genotypes share approximately 60% sequence identity. Based on phylogenetic analyses of the ORF5 sequences of the PRRSV strains isolated from different countries around the world, type 2 PRRSV is mainly classified into nine distinct lineages [8,9,10]. Each lineage can be further divided into several sub-lineages. The dominating type 2 PRRSV lineages in China include lineages 1, 3, 5 and 8 [8]. PRRSV was first reported in China in 1995, and the representative PRRSV strain, CH-1a (sub-lineage 8.1), was found to display mild pathogenicity [11]. In 2006, a large outbreak of PRRS caused by a highly pathogenic PRRSV (HP-PRRSV) represented by the JXA1 strain (sub-lineage 8.7) emerged in China and affected over 20 million pigs, causing huge economic losses to the swine industry [12,13].

The lineage 3 PRRSV, represented by the QYYZ strain, has mainly circulated in southern China since it was found in 2010 [14]. The lineage 1 PRRSV, represented by the NADC30 strain, was first isolated in the US in 2008. The NADC30-like PRRSVs represented by the JL580 strain were reported in China in 2015 [15,16]. These strains are prone to recombine with the circulating PRRSV strains in China, generating new recombinant viruses with altered antigen profiles, tissue tropisms and virulence. The currently used commercial PRRSV vaccines can provide partial protection against the NADC30-like strains [17,18]. The NADC30-like strains have spread widely in China since 2013 and have caused huge economic losses to the swine industry [15,16]. 

Recently, NADC34 PRRSV, which was classified as sub-lineage 1.5, was first reported, causing reproductive disorders in sows and a high mortality rate in piglets in the USA [19,20]. The virus then spread to Peru and caused PRRS outbreaks in many affected farms [21]. NADC34-like PRRSVs have been reported in the Heilongjiang, Liaoning, Henan and Fujian provinces of China since 2017 [22,23,24,25]. These were found to have the highest nucleotide similarities and similar patterns of 100-aa deletions in the nsp2 to the IA/2014/NADC34 strain [22,24,25]. However, unlike the IA/2014/NADC34 strain, which is highly pathogenic to piglets, the NADC34-like PRRSV strains isolated in China only displayed mild clinical signs under experimental conditions [23,26]. Most NADC30-like PRRSVs isolated in China were found to recombine with wild-type field PRRSV strains, generating new PRRSVs with different phenotypes [15,27].

Several recently published studies showed that some early reported Chinese NADC34-like strains did not contain recombinant events and were most similar to the IA/2014/NADC34 strain [22,24]. Novel PRRSV variants with evidence of recombination between viruses in the JXA1-like, NADC30-like and QYYZ-like strains and the emerging lineage 1.5 (NADC34-like) PRRSV have also been reported [28,29], but little is known regarding the pathogenesis of the PRRSV recombinants originating from NADC34-like and circulating strains. In the present study, we report the isolation and determination of the full-length genomes of three recombinant PRRSV strains derived from NADC34-like, NADC30-like and HP-PRRSV-like strains. The pathogenicity of two of the three recombinant viruses was also evaluated in piglets. 

## 2. Materials and Methods

### 2.1. Clinical Samples, Cells and Virus Isolation

Sera were collected from piglets showing dyspnea in swine-raising farms in Guangxi Province, southern China. The sera were confirmed as positive for PRRSV by RT-PCR. To isolate PRRSVs, the lung suspensions or serum samples were filtered through a 0.22 μm syringe filter (Millipore, Billerica, MA, USA). MARC-145 cells were grown as a monolayer in modified Eagle's medium (MEM) supplemented with 10% heat-inactivated fetal calf serum (FBS) and seeded in 6-well plate. The filtered samples were inoculated into the cells, and cytopathic effects (CPEs) were observed over 3–6 days. The supernatants from the MARC-145 cell were passaged several times. The isolated viruses were purified by end-point dilutions and plaque formation three times. 

### 2.2. Immunofluorescence Assay 

The MARC-145 cells in the 6-well plates were inoculated with the isolated PRRSV strains. After 1 h of incubation, the cells were washed three times with PBS and then fixed with cold acetone. The acetone was removed, and the cells were then blocked with 5% bovine serum albumin. A monoclonal antibody against PRRSV N protein (SDOW17, Rural Technologies, Inc, Brookings, SD, USA) was added to the cells and incubated for 1 h. The cells were washed three times with PBS and then incubated with goat anti-mouse IgG H&L, (Alexa Fluor® 488, Invitrogen, San Jose, CA, USA) secondary antibody for 1 h. Finally, the cells were washed three times with PBS and observed under a fluorescence microscope while in the PBS.

### 2.3. Viral RNA Extraction, Reverse Transcription and Complete Genome Determination

The viral RNA of the PRRSV strains was isolated from the sera using a Viral RNA Mini Kit (Axygen Biosciences, Union City, CA, USA) according to the manufacturer’s instructions. PRRSV RNA was measured by using a RT-PCR, as described previously [30,31]. For the whole genome amplification, the viral RNA was reverse transcribed into cDNA using a Prime Script Reverse Transcriptase (TaKaRa, Japan). Twelve segments covering the whole genome were amplified by PCR using the Prime STAR GXL DNA Polymerase kit (Takara, Kyoto, Japan). The primers used for the amplification of the whole genome are listed in Supplementary Table S1. The amplicons were gel-purified with an E.Z.N.A.TM Gel Extraction Kit (OMEGA, Norcross, GA, USA) and then cloned into the pMD18-T vector (TaKaRa, Dalian, China), following the manufacturer’s instructions. The positive clones were sequenced by using primers T7 or T3 (HuaDa Gene Inc., Guangzhou, China). The whole genomic sequences of the three PPRSV strains were assembled using the SeqMan program of DNAstar software, version 7.0, and then deposited in the GenBank database under the respective accession numbers: OK486522(GXFCG20210401), OK486523(GXQZ20210403) and OK486524(GXNN20210506).

### 2.4. Sequence Comparison and Evolutionary Analysis

The MegAlign program in DNAstar 7.0 software (DNASTAR Inc., Madison, WI, USA) was used to analyze the differences between the nucleotide and amino acid sequences from the three PRRSV strains reported in this study and some representative strains. MEGA 6.0 software with the neighbor-joining method was then used to perform the evolutionary analysis of these strains. Bootstrap values were estimated for 1000 replicates. The detailed information of the selected PRRSV reference strains is shown in Supplementary Table S2.

### 2.5. Recombination Analysis

The Recombination Detection Program (RDP) v4.66 with seven different algorithms (RDP, BootScan, SiScan, Chimaera, GENECONV, MaxChi and 3Seq) was used for recombination analysis. The recombinant events in the PRRSV genomes were confirmed by a Bootscan analysis in Simplot software (v3.5.1, JHK University, Baltimore, MD, USA), with the default parameters. These putative recombination events were further confirmed by constructing phylogenetic trees for each of the sequence regions.

### 2.6. Pathogenicity of the Recombinant PRRSV Strain in Piglets

The animal experiments were carried out in accordance with the guidelines issued by the Animal Care & Welfare Committee of Guangxi University (GXU2019-043). Fourteen 28-day-old piglets of both sexes were diagnosed as negative for PRRSV by RT-PCR and a PRRSV antibody ELISA kit (JNT PRRSV-Ab ELISA kit, Beijing, China). The piglets were randomly allocated into a control group (*n* = 4), a GXFCG20210401-challenged group (*n* = 5) and a GXQZ20210403-challenged group (*n* = 5). PRRSV-challenged piglets were inoculated intramuscularly and intra-nasally with 2 mL (10^4^TCID50/mL) at each site. The piglets in the control group were inoculated intramuscularly and intra-nasally with 2 mL DMEM.

All of the piglets were monitored daily for clinical signs of disease, and their rectal temperatures were recorded daily. The body weights of each piglet were measured at 0, 7 and 14 days post-infection (dpi). Nasal and rectal swabs were collected for detection viral RNA shedding by RT-PCR [32]. Sera were collected at 0, 3, 5, 7, 10 and 14 dpi for viremia detection by using qPCR. The PRRSV antibodies in sera were measured using an ELISA kit (JNT PRRSV-Ab ELISA kit, Beijing, China). All the piglets were subjected to necropsy after euthanasia at 14 dpi. The tissue samples, including the thymus, lymph node, tonsil, brain sections, heart, spleen, liver, lung, kidney, small intestine and stomach, were collected from each piglet for the detection of viral loads by qPCR [32].

### 2.7. Hematoxylin-Eosin and Immunohistochemistry

The tissue samples of the thymus, lymph node, tonsil, brain sections, heart, spleen, liver, lung, kidney, small intestine and stomach were dehydrated and then fixed in 4% paraformaldehyde for hematoxylin-eosin staining. Samples of the lung tissue were used for immunohistochemical studies. A monoclonal antibody (SDOW17, Rural Technologies, Inc, Brookings, SD, USA) for the PRRSV N protein and HRP-conjugated goat anti-mouse IgG (H+L) was used for immunohistochemistry staining. The stained sections were observed under a microscope (Nikon E100). According to previous report [18], the microscopic pathological lesions and the intensity of the IHC signal after the staining of the lung tissues in each group were graded and scored.

### 2.8. Statistical Analysis

In this study, t-tests and multiple comparisons were performed to compare the differences in the means of changes in rectal temperatures, body weights, antibody levels and virus copy numbers in each group of piglets. All the data in this report are shown as the means ± SDs. Data analysis was conducted using GraphPad Prism 5 software (San Diego, CA, USA). Statistical analyses were performed with the two-tailed, unpaired Student's t-test. When multiple comparisons were performed, one-way ANOVA followed by Tukey's test or one-way ANOVA with Dunnett's test were performed. The data presented met the assumptions of the statistical test employed. Differences were regarded as statistically significant at *p* < 0.05 and as extremely significant at a value of *p* < 0.01.

## 3. Results

### 3.1. Isolation and Identification of PRRSV Strains

**Figure 1:**
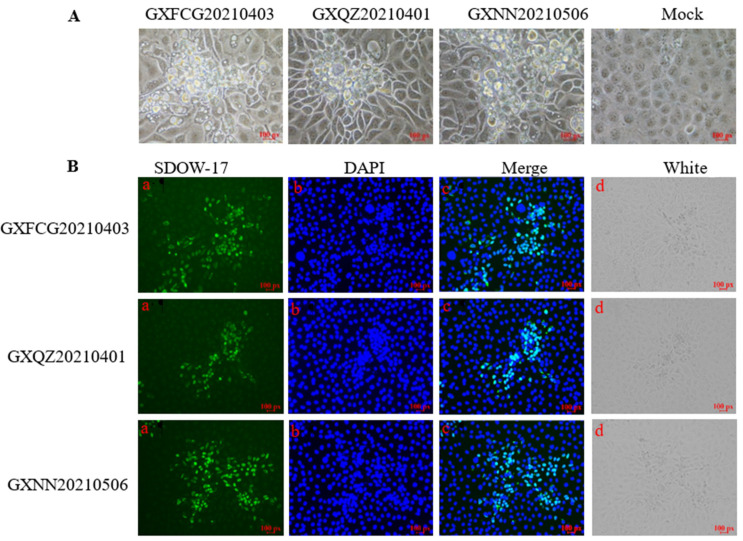
(**A**) Cytopathic effect (CPE) in MARC145 cells at 72 h post-infection with GXFCG20210403, GXQZ20210401, GXNN20210506 and Mock, respectively. Scale bar = 100×. (**B**) The existence of GXFCG20210403, GXQZ20210401 and GXNN20210506 was confirmed by IFA staining using a PRRSV-specific N protein monoclonal antibody, SDOW-17, as shown in (**a**). Nuclei were counterstained with DAPI and can be visualized in blue (**b**). The combined images of staining with N-mAb and DAPI are shown in (**c**). The photomicrograph (**d**) shows the infection state of MARC145 cells after fixation under white light irradiation. Scale bar = 100×.

A distinct CPE in MARC145 cells inoculated with sera from diseased piglets showing respiratory syndrome was observed. Three PRRSV strains were designated as GXFCG20210401, GXQZ20210403 and GXNN20210506, respectively. The CPEs were characterized by cell rounding and shrinkage. Some cells were detached from the 6-wells plate surface (Figure 1). IFA showed specific PRRSV N protein expression in the MARC-145 cells inoculated with GXFCG20210401, GXQZ20210403 and GXNN20210506, indicating that the isolates propagated in the MARC-145 cells (Figure 1).

### 3.2. Complete Genome Sequence of PRRSV Strains 

Excluding the poly A tail, 15,021 nt, 15,019 nt and 15,022 nt were determined for GXFCG20210401, GXQZ20210403 and GXNN20210506 (GenBank accession number: OK486522, OK486523 and OK486524), respectively. Genome sequence alignments of these PRRSV reference strains (JXA1, CH-1a, CHSX1401, NADC30, VR-2332, NADC34 and QYYZ) were carried out. The full-length genome of the three PRRSV isolates shared 82.5–88.7% identity with the reference PRRSV strains and showed the highest identities with NADC30 (88.7, 88.6 and 88.3%, respectively; Supplementary Table S3).

The UTR and the amino acid sequence coded by each ORF of the three PRRSV isolates were then compared with those of the reference strains. As shown in Supplementary Table S3, the 5′UTR of the PRRSV isolates GXFCG20210401 and GXNN20210506 exhibited the highest identities (97.4 and 96.8%, respectively) with that of JXA1, and the 5′UTR of GXQZ20210403 showed the highest identity (96.8%) with that of NADC30-like strains. The 3′UTR of the PRRSV isolates (GXFCG20210401, GXQZ20210403 and GXNN20210506) showed the highest identities (97.3, 96.6 and 97.3%, respectively) with NADC30. The nsp1 and nsp9 of GXFCG20210401 and GXNN20210506 displayed the highest nucleotide (91.2–92.0%) and amino acid (91.6–97.7%) similarities with those of the JXA1 strains. The nsp2-nsp8, nsp10-nsp12 and ORF6 of GXFCG20210401 and GXNN20210506 displayed the highest nucleotide (83.4–96.3%) and amino acid (80.1–100.0%) similarities with those of the NADC30-like strains. The ORF2a-ORF5 and ORF7 displayed the highest nucleotide (93.3–96.8%) and amino acid (93.4–98.6%) similarities with the NADC34 strain.

The nsp1, nsp4*–*5 and nsp7*–*8 of GXQZ20210403 exhibited the highest nucleotide (91.6*–*95.1%) and amino acid (92.9*–*97.5%) similarities with those of the JXA1 and CH1A strains. The nsp2-nsp3, nsp9-nsp12 and ORF6-7 of GXQZ20210403 displayed the highest nucleotide (87.8*–*96.0%) and amino acid (83.7*–*98.0%) similarities with those of the NADC30 and CHSX1401 strains. The ORF2a-ORF5 of GXQZ20210403 displayed the highest nucleotide (94.5*–*97.4%) and amino acid (93.0–98.6%) similarities with the NADC34 strain. These results indicate that the three PRRSV isolates may be made up of mosaic isolates that originated from the predominant circulating PRRSV strains and the emerging NADC34-like strains (Table 1).

Nsp2 is the most variable protein in PRRSVs and contains different patterns of amino acid deletions and insertions. The sequence alignments of the PRRSV strains showed that the three strains had same discontinuous deletions of 111 + 1 + 19 amino acids in the nsp2 region when compared with VR2332 (Supplementary Table S1a). These deletions, which were considered to be a genetic marker, also occurred in the genome of the NADC30-like strains.

GP5 is the most enriched and variable envelope glycoprotein of PRRSVs. An analysis of the GP5 sequences of the three isolates showed that the N-terminal signal peptide and the C-terminal cellular epitope regions were more variable when compared to the VR2332 strain, while the amino acids at positions 26–27 in the decoy epitope were changed (Supplementary Table S1b). An L41S amino acid substitution was present in the PNE region of the GXFCG20210401 strain. In addition, the K59S and R151K substitutions occurred in the three isolates at positions 59 and 151, and these are thought to be associated with viral virulence. Like other known NADC34-like strains, the three isolates contain five predicted glycosylation sites in GP5. 

### 3.3. Phylogenetic Tree

**Figure 2:**
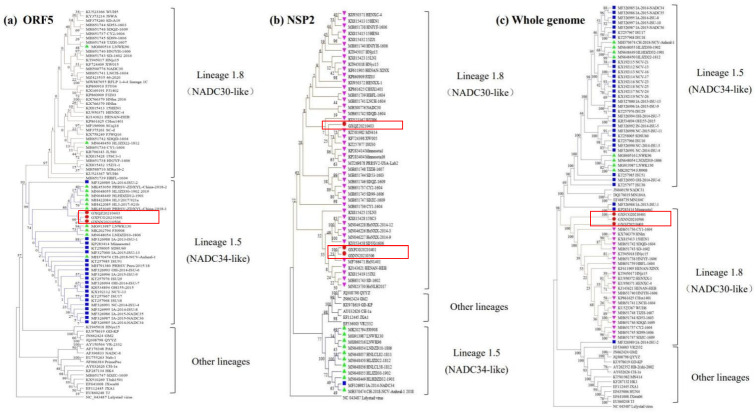
(**a**) Genetic evolutionary tree of the ORF5 gene. (**b**) Genetic evolutionary tree of the nsp2 gene. (**c**) Genetic evolutionary tree of the full-length genome. The categorization of the sub-lineage was based on the description by Shi et al. [10]. The phylogenetic tree was constructed using the Mega 6.0 distance-based neighbor joining method, with a total of 1000 replicates. The virus strains obtained in this study are marked with red circles and red boxes. The Chinese NADC34-like isolates are marked with green triangles. The American and Peruvian 1-7-4 PRRSV strains are labelled with blue squares. The NADC30-like isolates are marked with pink triangles. In the evolutionary tree branches, the NADC30-like strains are indicated by brown lines, the NADC34-like strains are indicated by blue lines and the other lineages and sub-lineages are indicated by black lines.

The phylogenetic tree based on the ORF5 showed that the PRRSV strains could be divided into nine lineages. The three isolates belonged to sub-lineage 1.5, which is represented by IA/2014/NADC34 and NADC34-like strains isolated in China, namely, CH/2018/NCVAnheal-1, HLJDZD32-1901 and HLJZD30-1902 (Figure 2a). The phylogenetic tree based on the nsp2 and the complete genome showed that the PRRSV isolates belonged to sub-lineage 1.8, together with the reference strain and the NADC30 and NADC30-like strain, CHSX1401 (Figure 2b,c). These trees suggest that the three PRRSV isolates may have originated from older NADC30-like stains as well as the emerging NADC34-like strains.

### 3.4. Recombination Analysis

**Figure 3:**
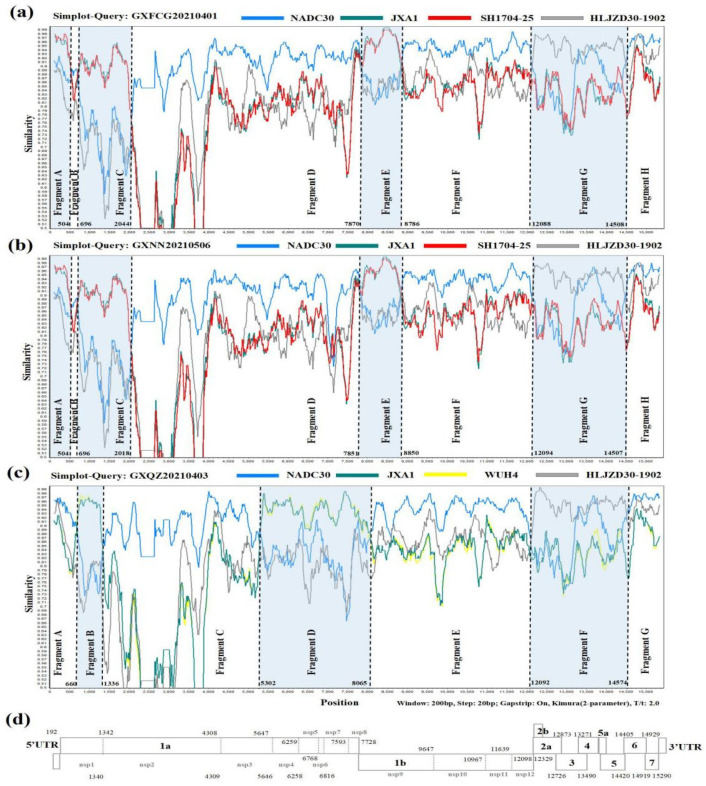
Simplot was utilized to compare the similarities of GXFCG20210401 (**a**), GXNN20210506 (**b**) and GXQZ20210403 (**c**) to potential parental viruses, respectively. Recombinant breakpoints are displayed as black virtual lines. The background color of the primary parental areas (NADC30-like) is in white, whereas that of the minor parental areas (HP-PRRSV- and NADC34-like) is in blue. NADC30, JXA1, SH1704-25 and HLJZD30-1902 are depicted by blue, green, red and grey colorations in the plotted similarity curves, respectively. (**d**) The whole genome structure of PRRSVs, which shows the location of the 10 open reading frames and non-structural proteins.

**Figure 4:**
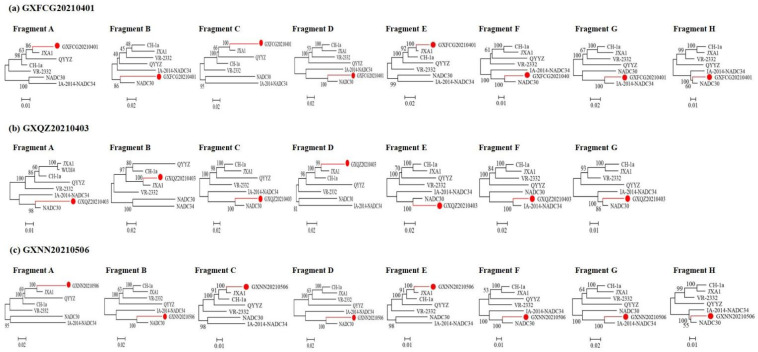
The isolates that were identified in the evolutionary tree are marked with red lines and red circles. The phylogenetic analysis of the different fragments of strain GXFCG20210401 is shown in (**a**). The phylogenetic analysis of the different fragments of strain GXQZ20210403 is shown in (**b**). The phylogenetic analysis of the different fragments of strain GXNN20210506 is shown in (**c**).

The SimPlot and RDP4 software packages were used to identify possible recombination events in the whole genomes of the three novel PRRSV strains. The result of the RDP4 software showed that the whole genomes of GXFCG20210401, GXQZ20210403 and GXNN20210506 showed high degrees of certainty according to the results of at least five detection methods (Supplementary Table S4). A similarity plot analysis showed that the GXFCG20210401 genome had seven recombination breakpoints (positions of alignment) that were located in nsp1α (nt 504 and nt 696), nsp2 (nt 2044), nsp9 (nt 7870 and nt 8786), nsp12 (nt 12088) and ORF6 (nt 14508) (Figure 3a). The breakpoints divided its genome into eight fragments. Phylogenetic analysis showed that fragment A (5'UTR-nsp1α, nt 1–504), fragment C (nsp1α-nsp2, nt 696–2044) and fragment E (nsp9, nt 7870-8786) were clustered with the JXA1-like and Ch1a-like strains. Fragment B (nsp1α, nt 505-695), D (nsp2-nsp9, nt 2045-7869), F (nsp9-nsp12, nt 8787-12087) and H (ORF6-3'UTR, nt 14509-3'UTR) were closely associated with the NADC30-like strain, and fragment G (nsp12-ORF6 nt 12088–14508) was closely related to the NADC34-like strain (Figure 4a).

The GXNN20210506 strain shares a similar recombination pattern to the GXFCG20210401 strain (Figure 3b and Figure 4c). For GXQZ20210403, six recombination breakpoints located in nsp1α (nt 660), nsp1β (nt 1336), nsp3 (nt 5302), nsp9 (nt 8065), nsp12 (nt 12092) and ORF6 (nt 14574) were identified in its genome (Figure 3c). The breakpoints in GXQZ20210403 divided its genome into seven fragments. The phylogenetic trees showed that fragments B (nsp1α-nsp1β, nt 660-1336) and D (nsp3-9, nt 5302-8065) were closely related to the JXA1-like strain. Fragments A (5′UTR-nsp1α, nt 1-659), C (nsp1β-nsp3, nt 1337-5301), E (nsp9-nsp12, nt 8066-12091) and G (ORF6-3′UTR, nt 14575-3'UTR) were closely associated with the NADC30-like strain. Fragment F (nsp12-ORF6 nt 12092–14574) was clustered with the NADC34-like strain (Figure 4b). These results indicated that the GXFCG20210401, GXQZ20210403 and GXNN20210506 strains were likely to have originated from multiple recombination events among the JXA1-, NADC30- and NADC34-like strains.

### 3.5. Observations of Clinical Signs

**Figure 5:**
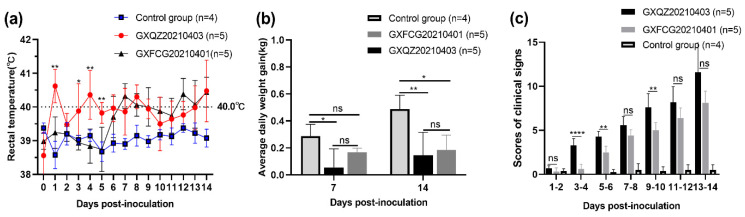
(**a**) The rectal temperatures of piglets inoculated with GXFCG20210401, GXQZ20210403 and DMEM media. The clinical fever cut-off value was set at 40.0 °C. (**b**) The average daily weight gain of the inoculated piglets during each week in the challenge trial. (**c**) The clinical sign scores for inoculated piglets. The values are expressed as means ± standard deviations, the bars represent the average values and error bars indicate standard deviations. Asterisks indicate the significant differences between the GXFCG20210401 and GXQZ20210403 infection groups when compared to the controls (* *p* < 0.05; ** *p* < 0.01; **** p < 0.0001; ns, no significant difference).

In order to explore the pathogenicity of recombinant PRRSV containing HP-PRRSV-, NADC30- and NADC34-like fragments, the PRRSV strains GXFCG20210401 and GXQZ20210403 were selected for pathogenicity tests in 4-week-old piglets. Compared with the piglets in the control group, which showed a normal physiological temperature and good health throughout the entire study period, the piglets infected with GXQZ20210403 showed body temperatures of over 40 °C on 1, 4, 8 and 14 dpi (Figure 5a). Clinical symptoms such as a cough, runny nose, anorexia and redness and swelling of the conjunctiva began to appear within 3–5 dpi, and severe symptoms characterized by the inflammatory adhesion of the conjunctiva, tachypnea, unstable standing, drowsiness and dyspnea were observed from 7 to 14 dpi. Two piglets infected with GXQZ20210403 were extremely emaciated at 13 dpi, one of which died and the other showed extreme asthenia. Piglets infected with GXFCG20210401 showed a febrile response (39.7 °C) at 6 dpi and a peak of 40.4 °C at 14 dpi. From 6 to 14 dpi, the piglets showed an abnormally high physiological body temperature (≥ 39.7 °C) for nine consecutive days. (Figure 5a). Piglets infected with GXFCG20210401 exhibited milder clinical symptoms. These included coughing, redness of the conjunctiva, anorexia, lethargy, tachypnea and slow growth, which were observed at 6–14 dpi, but all the piglets survived for the duration of the study. To reflect the pathogenicity of the two recombinant PRRSV strains, we conducted blind evaluation scores on all the piglets from different groups. From 1 to 14 dpi, the mean clinical scores of the GXQZ20210403- and GXFCG20210401-infected groups were significantly higher than those of the control group, and the difference between the GXQZ20210403- and the GXFCG20210401-infected groups was highly significant (*p* < 0.01; Figure 5c). 

### 3.6. Antibody Levels, Viremia and Virus Load in Tissues and Swabs

**Figure 6:**
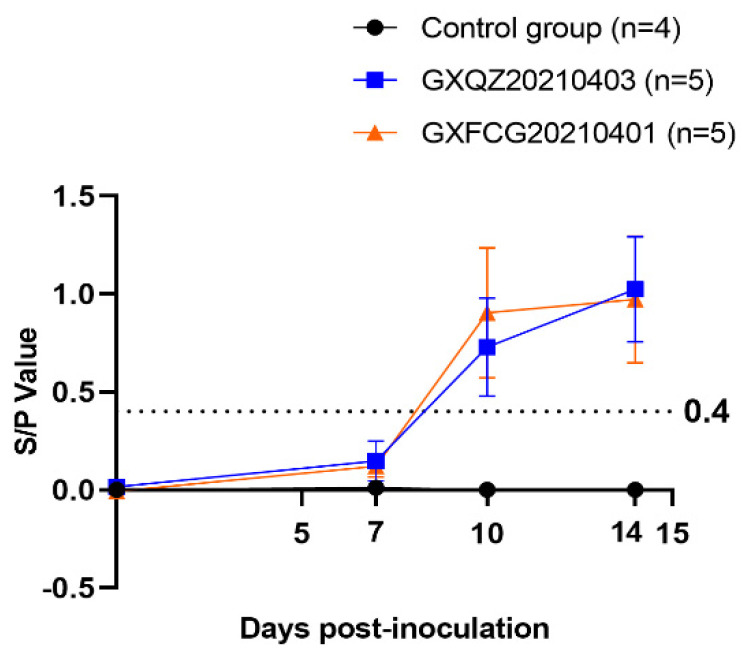
PRRSV-specific N protein antibodies in sera from pigs challenged for different days after infection. The cut-off value for seroconversion was set at a sample-to-positive (s/p) ratio of 0.4. The values are expressed as means ± standard deviations.

**Figure 7:**
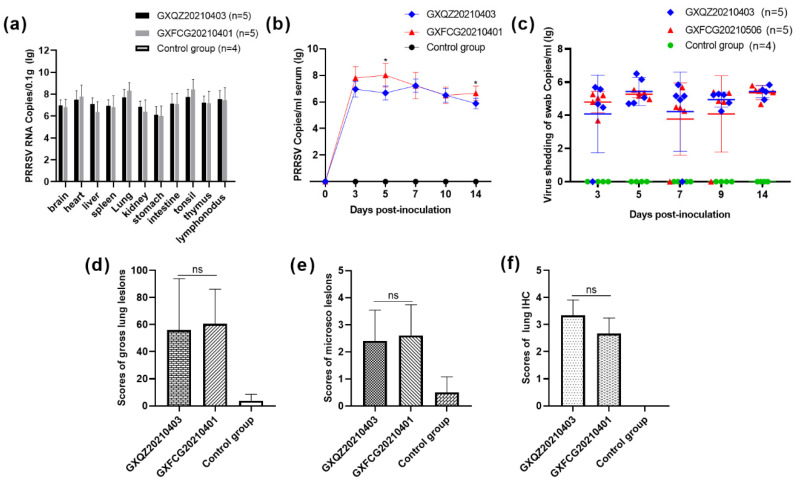
(**a**) Determination of RNA copy numbers of PRRSVs for different organs of the piglets in each group, as measured by qRT-PCR. (**b**) The number of PRRSV RNA copies in the sera of pigs on different days after the challenge were quantified by qRT-PCR. (**c**) The nasal and rectal swabs were collected on different days to detect virus shedding from piglets in each group. (**d**) The scores of lung gross lesions were graded based on the percentage of the lung areas affected. (**e**) The scores of lung microscopic lesions were graded based on the distribution and severity of interstitial pneumonia. (**f**) The scores of the numbers of PRRSV-positive cells in the lungs. The values are expressed as means ± standard deviations, the bars represent the average values and error bars indicate standard deviations. Asterisks indicate the significant differences between the GXFCG20210401- and GXQZ20210403-infected groups when compared to the controls (* *p* < 0.05; ns, no significant difference).

A commercially available JNT ELISA kit was employed to measure PRRSV N protein-specific antibodies in pig sera at 0, 7, 10 and 14 dpi. As shown in Figure 6, all piglets in both challenged groups remained negative for PRRSV antibodies at 7 dpi (<0.4) and showed serum conversion at 10 dpi and 14 dpi. PRRSV-specific antibodies in the control group remained negative throughout the experimental period. Virus titers in serum samples collected at 0, 3, 5, 7, 10 and 14 dpi from the GXFCG20210401- and GXQZ20210403- infected groups reached peak levels at 5 and 7 dpi, respectively (Figure 7b). Viraemia in serum samples from the control group remained negative throughout the experimental period. After the euthanasia of each group of piglets, organ tissues were collected from each group and were examined for viral load (Figure 7a). The results of the viral load in all organs showed that the virus was detected in all the tissues of the two infection groups, with the highest viral load in the heart, lungs, tonsils and lymph nodes. Viral RNA could not be detected in the control piglet tissue samples.

In addition, nasal swabs were collected at 0, 3, 5, 7, 9 and 14 dpi for the determination of virus shedding. Viral titers in the nasal swabs from piglets infected with GXQZ20210403 ranged from 1.22 × 10^5^ to 9.81 × 10^5^ copies/mL within 3*–*14 dpi and peaked at 5 dpi with a titer of 9.81 × 10^5^ copies/mL. In the GXFCG20210401-inoculated group, high levels of shedding titers could be detected within 7*–*14 dpi and peaked at 14 dpi with a titer of 3.06 × 10^5^ copies/mL (Figure 7c). No virus shedding was detected in the control group during the whole experiment. 

### 3.7. Macroscopic and Histopathological Analysis

**Figure 8:**
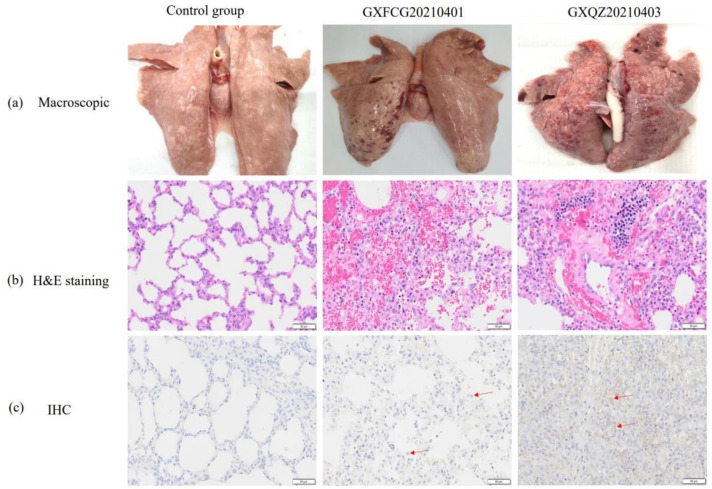
(**a**) The macroscopic lesions in the lungs of piglets within each group. (**b**) Representative images of the lung tissues stained with H&E. (**c**) Specific brown signals of PRRSV were observed in the lungs of pigs in the GXFCG20210401 and GXQZ20210403 challenged groups (marked by the red arrows).

The lung lesions of piglets in both infected groups were characterized by symptoms of interstitial pneumonia, emphysema, multifocal pulmonary hemorrhage, petechiae and other typical lesions of PRRSVs (Figure 8a). Some areas of the parenchymal organs were earthy yellow with an uneven coloration, especially in the kidneys. In addition, the lymph nodes in the mediastinal and inguinal regions showed enlargement and hyperplasia, and punctate hemorrhaging had occurred in the lymph nodes of some piglets (Supplementary Figure S1). In contrast, the piglets in the control group showed normal morphology and staining of all tissues, and the organs were without significant lung pathology. The mean lesion scores for the infected pigs were 56 and 60.6% for GXQZ20210403 and GXFCG20210401, respectively (Figure 7d). The average score of lung injury in the challenged group was significantly higher than that in the control group.

Histopathologically, lung slices from both infected groups showed the activated proliferation of type II cells in the alveoli, diffuse and multifocal interstitial pneumonia, the thickening of connective tissue in the alveolar walls and alveolar septa and bronchial hyperplasia. This was accompanied by a large infiltration of lymphocytes and plasma cells into the interstitial layer of the lungs (Figure 8b). In the parenchymal organs, the piglets in both infected groups showed some degree of degenerative changes. For example, vacuolar degeneration was found in the livers of both infected groups, with lymphocytic infiltration within the lobules and the hepatic portal vein areas (Supplementary Figure S2). That was most evident in the renal corpuscles of the renal sections, which showed granular degeneration, mild damage to the basement membrane, a small amount of lymphocytic infiltration in the renal interstitium and multifocal hemorrhage in the renal medullary areas.

In the lymphoid organs (the mediastinal and inguinal lymph nodes and the spleen), the lesions were characterized by follicular and paracortical hyperplasia, especially the active proliferation of reticular cells, and in the spleen, the were characterized by the incomplete and severe disintegration of splenic vesicles, the laxity of splenic trabeculae and a marked decrease in lymphocytes (Supplementary Figure S2). The lung slices were subjected to immunohistochemical staining, and both GXQZ20210403 and GXFCG20210401 strains induced positive brownish-red staining of the epithelial cells and macrophages in the piglet lungs. Similar lung microscopic lesions and IHC staining scores were observed in all of the challenged groups (Figure 7e,f), with no statistical differences (*p* > 0.05). In contrast, no brownish-red cells were identified in the piglet lung tissues of the control group (Figure 7f and Figure 8c). 

## 4. Discussion

As one of the most mutation-orientated RNA viruses, there are a wide range of PRRSV subtypes created by its variability that are prevalent throughout the world. This has resulted in huge economic losses for the swine industry in numerous countries [12,13,20,21]. In China, since the HP-PRRS outbreak in 2006, the PRRSV population has undergone a phase of alternating dominance between HP-PRRSV-like and NADC30-like strains and may now be undergoing a further change [12,13,16]. According to Xu et al., the combined prevalence rate of NADC34-like strains in 14 provinces in China has increased from 11.5 to 28.6% in 2020–2021, indicating a possible epidemic trend [29].

In this study, three novel NADC30-like strains were isolated—namely, GXFCG20210401, GXQZ20210403 and GXNN20210506—from serum samples that had recombinant fragments of NADC34- and HP-PRRSV-like strains from three different cities in Guangxi Province. Comparative analysis showed that the isolates showed extensive genetic variability with representative strains of NADC30, VR-2332, QYYZ, CH-1a and JXA1. The nucleotide similarities of the whole genomes of the three isolates GXFCG20210401, GXQZ20210403 and GXNN20210506 with PRRSV representative strains were low, only sharing the highest identities with the NADC30 strains (88.7, 88.6 and 88.3%, respectively). A comparison of the UTR and each ORF region of the genomes of the three PRRSV isolates with those of the reference strains showed that most parts of the structural coding ORFs displayed the highest nucleotide and amino acid similarities with NADC34-like strains. The other region of the three isolates showed the highest nucleotide and amino acid similarities to the JXA or NADC30 strains. The nsp2 region is the largest non-structural protein in the genome, and it can harbor a variety of mutations such as deletions, recombination and insertions [4,33,34]. When compared to strain VR-2332, there were three major regions of deletions (111 + 1 + 19 aa) in the nsp2 of the three PRRSV strains. These deletions, which were considered as genetic markers of the virus, also occurred in the genome of the NADC30-like strains [25,35].

GP5 is the main capsule membrane structural protein, and it is also highly variable in PRRSVs. It has the important function of inducing the production of neutralizing antibodies. Usually, GP5 possesses two to five potential N-linked glycosylation sites in the ectodomain in the field strains of these viruses [36,37]. The three novel isolates reported in this study have five predicted potential N-linked glycosylation sites that are similar to the NADC34 and NADC34-like strains isolated from China [38]. The presence of 7–9 amino acid substitutions in the GP5 region was detected in the three isolates, and the most variable regions were the N-terminal signal peptide and the C-terminal cellular epitope regions. In addition, amino acid substitutions were present in both the PNE and decoy regions. The gained N-glycosylation sites and the amino acid substitutions in the specific antigenic sites may allow these recombinant viruses to escape the immunity afforded by commercially used vaccines [39].

The three PRRSV isolates were found in the phylogenetic tree to be classified together with NADC34 into the branch of lineage 1.5 and were located in the independent branch formed by the NADC34-like strains found in China. According to the evolution of the whole genome, the NADC34-like strains prevalent in China can be further divided into two subgroups: A and B [40]. The three isolated strains in this study were clustered in the A subgroup represented by HLJDZD32-1902 in China, suggesting that the ORF5 sequence of the parent strain was closely related to the representative strains of this subgroup. However, phylogenetic trees based on the nsp2 region and the whole genome showed that all three isolates belonged to lineage 1.8, as represented by NADC30. This indicates that there may have been recombinant events between the three isolated strains and the emerging NADC34-like strains. In addition, this reflects the limitations of epidemiological investigations based on the evolutionary tree of only the ORF5 region, which can lead to certain recombinant strains being ignored. The above comparative analysis and phylogenetic trees analysis imply that the three isolates were recombinants with complex recombination events in the genome that originated from the prevalence of NADC30-like strains, with the participation of HP-PRRSV-like and domestic NADC34-like strains.

According to previous reports [30,41], frequent recombination events occurred between the strains of lineage 1, represented by NADC30, as well as other lineages. A whole-genome-recombination analysis of 13 isolated NADC30-like strains revealed that 10 of their genomes were replaced with various gene fragments of different lengths from other PRRSV strains [30]. The early circulating strains, FJZ03 and FJWQ16, are from lineage 1, and these have recombined with the recombinant fragment from the attenuated vaccine of lineage 8 of HP-PRRSV [41]. The 15JX1, 15HEN1 and 15SC3 strains are all the results of recombinant events that occurred between the NADC30-like and JXA1-R strains [34]. The frequent recombination of lineage 1 PRRSVs can occur between different lineage strains and even between three different lineages (lineages 8, 5 and 1), such as SCcd17 and SDhz1512 [42,43]. The recombinant analysis of the three isolated strains in our study showed that all three strains were recombinant variants resulting from different degrees of mosaicism with HP-PRRSV-like strains such as the WUH4-, SH1704-25-, JL-04/12-, NADC34- and HLJZD30-1902-like strains. These are based on the domestic NADC30-like strains such as FJZ03 and FJWQ16, which acted as the main source.

Studies by Yu et al. and Zhao et al. [33,34] reported that the strains from lineage 1 and 8 strains were more prone to recombination events than others. They found that the high-frequency recombination regions were mainly distributed between nsp2, nsp9 and ORF2-ORF3. In the present study, we found recombination events in the nsp9 regions of all three strains, suggesting an important role for nsp9 in the recombination mechanism of PRRSVs. In addition, the Chinese HLJZD30-1902-like strain was involved in the genomic recombination of all three novel strains, suggesting that the NADC34-like strain has already been endemic in southern China and was co-circulating with the predominant local PRRSV strains.

Since Zhang et al. first identified and reported the presence of NADC34-like strains in China in 2017, more than twenty strains associated with NADC34-like strains have been discovered in at least 10 provinces [25,29,40]. It is worth noting that, since NADC34-like strains are in the same lineage as NADC30, the NADC34-like strains may also undergo frequent mutation and recombination events, as the NADC30-like strains do. In China, the NADC30 mosaic recombinant strains, which are associated with the vaccine and local predominant strains, have become the major prevalent strains of PRRSVs [34,41]. However, the increasing prevalence of the NADC34-like strains will further exacerbate the existing complex recombination situation, and these have a strong potential to replace the NADC30- and HP-PRRSV-like strains.

The altered biological properties of PRRSVs are mainly motivated by recombination events, as evidenced by marked changes in antigenicity and cell tropism, thereby reducing the protective immunity induced by existing vaccines [17,18,41]. It has been shown that the two NADC30-like PRRSV strains, JL580 and FJ1402, are recombinants of an HP-PRRSV-like strain (09HEN1/GD), and these were shown to be highly pathogenic in vivo [15,27]. Based on field observations, a NADC34 PRRSV strain was first reported to cause dramatic “abortion storms” in sow herds and a high mortality in piglets in the USA [20]. The NADC34-like PRRSVs were also reported to be associated with high abortion rates in sows (10 to 30%) as well as with a high mortality in suckling pigs (10 to 80%) in affected Chinese pig farms. Consistent with field observations, pigs infected experimentally with the NADC34 strains, such as IA/2014/NADC34, IA/2013/ISU-1 and IN/2014/ISU-5, had a persistent fever and retarded growth, and some of the infected pigs died late in the experiments [26,28,40]. In contrast, the Chinese NADC34-like PRRSV, HLJDZD32-1901, was found to be a milder pathogenic strain in piglets [23].

A recent study has shown that PRRSV recombinants, which originated from recombination events between NADC34- and QYYZ-like strains, can have a high pathogenicity to piglets [28]. Novel PRRSV variants with evidence of recombination between viruses in lineages 8 (JXA1-like) and 1.8 (NADC30-like) and the emerging lineage 1.5 (NADC34-like) have been reported [29], but very little is known regarding the pathogenesis of the PRRSV recombinants originating from NADC34-like and circulating strains. In the present study, the recombinant viruses GXQZ20210403 and GXFCG20210506, which contain a fragment of the NADC34-like gene, were evaluated for their pathogenicity in piglets. Pigs inoculated experimentally with GXFCG20210506 displayed mild clinical signs including a mild cough, anorexia, a slight increase in body temperature and retarded growth, and they all survived until the end of the experiment. In contrast, pigs inoculated with GXQZ20210403 displayed more severe clinical symptoms such as tachypnea, lameness, drowsiness and dyspnea, a persistent fever and retarded growth. One of the pigs in this group died, and another was unable to stand due to weakness late in the experiment. Each infected group had gross lesions, and the mean lesion scores for the infected pigs were 56 (GXQZ20210403) and 60.6% (GXFCG20210506). This was similar to what was found for the previously reported SDSU73 and IA/2014/NADC34 strains. Our results demonstrated that GXQZ20210403 was a higher pathogenic strain in piglets than GXFCG20210506.

## 5. Conclusions

In conclusion, we identified and characterized three novel recombinant variants of NADC34-like strains which formed between the dominant PRRSV-2 strains (NADC30- and HP-PRRSV-like) in Guangxi Province, southern China and confirmed that they were moderately pathogenic to piglets. It is worth noting that the recombinant strains came from three different cities in Guangxi Province, China, suggesting that the recombinant NADC34-like parental strains and the recombinant offspring strains may already be widely present and circulating around this area. This highlights the importance of constant monitoring and the ability to prevent the spread of NADC30- and NADC34-like strains in pig farms worldwide.

## Figures and Tables

**Table 1:** The predicted N-glycosylation sites of the PRRSV GP5 protein.

Predicted N-Glycosylation Sites of the PRRSV GP5 Protein		
Strains	N-Glycosylation Sites	Numbers
GXFCG20210401	N32, N33, N44, N51, N57	5
GXQZ20210403	N32, N33, N44, N51, N57	5
GXNN20210506	N32, N33, N44, N51, N57	5
IA/2014/NADC34	N32, N33, N44, N51, N57	5
NADC30	N34, N44, N51	3
CHsx1401	N33, N44, N51	3
JXA1	N30, N34, N35, N44, N51	5
QYYZ	N34, N44, N51	3
GM-2	N33, N44, N51	3
CH-1a	N34, N44, N51	3
VR2332	N34, N44, N51	3
FJ0908	N32, N33, N44, N51	4
LNWK96	N33, N34, N44, N51	4
LNWK130	N32, N34, N44, N51	4
HLHDZD32-1901	N32, N33, N44, N51	4
HLJZD22-1812	N32, N33, N44, N51	4
HLJZD30-1902	N30, N33, N44, N51	4
LNDZD10-1806	N30, N33, N44, N51	4
PRRSV-ZDXYL-China-2018-1	N32, N33, N44, N51, N57	5
PRRSV-ZDXYL-China-2018-2	N32, N33, N44, N51	4
CH-2018-NCV-Anheal-1	N32, N33, N34, N44, N51, N57	6
JS2021NADC34	N32, N33, N44, N51	4

## Data Availability

The complete genome sequences of the PRRSV strains GXFCG20210401, GXQZ20210403 and GXNN20210506 obtained in this study have been deposited in the GenBank under the acces-sion number OK486522, OK486523 and OK486524, respectively.

## Supplementary Materials

The following are available online at https://www.mdpi.com/article/10.3390/v14081695/s1, Figure S1. (A) Anatomical changes in the heart between the three groups, with the challenged groups showing slight steatosis. Figure S2. Heart, a small amount of inflammatory infiltration was present in the myocardial tissues of the challenged groups when compared to the control group. Table S1. Primers for amplification of the whole-length PRRSV genome. Table S2. The detailed information of selected PRRSV reference strains. Table S3. Comparison of ORFs and amino acids of three PRRSV isolates with representative strains of other lineages or sub-lineages. Table S4. Recombination events of PRRSV isolates GXQZ20210403, GXFCG20210401 and GXNN20210506 detected by RPD5 software.
