# Supplementary material for: The Emergence and Pathogenesis of Recombinant Viruses Associated with NADC34-like Strains and the Predominant Circulating Strains of Porcine Reproductive and Respiratory Syndrome Virus in Southern China
Source: Viruses. 2022 Jul 31;14(8):1695. doi: 10.3390/v14081695 (PMC9416154; doi:10.3390/v14081695)
Supplement: Supplementary file 1 [file viruses-14-01695-s001.zip › viruses-1826590-supplementary.pdf]

viruses MDPI

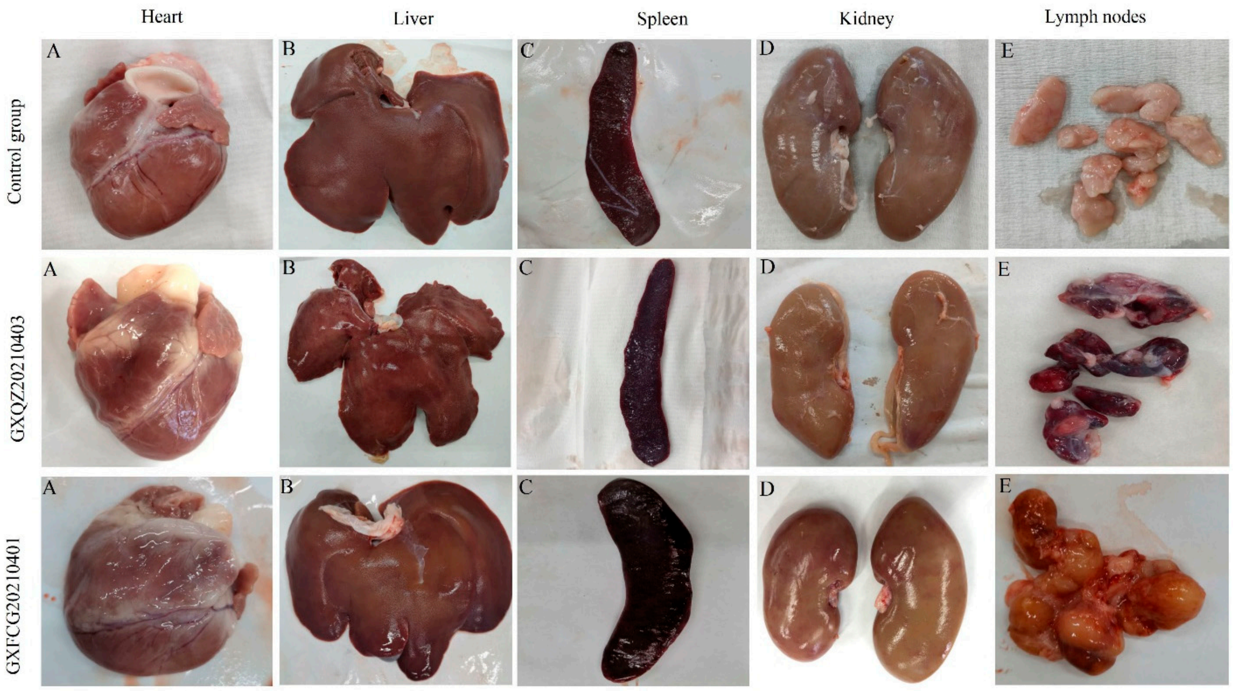

Figure S1. (**A**) Anatomical changes in the heart between the three groups, with the challenged groups showing slight steatosis. (**B**) Visual pathological changes in the liver between the three groups and yellow staining, petechiae and other changes are seen in the challenged group. (**C**) Visual pathological changes in the spleen between the three groups. (**D**) Visual pathological changes in the kidneys between the three groups, showing a distinct khaki coloration in the challenged group. (**E**) Visual pathological changes in the lymph nodes between the three groups with congestion, enlargement and other pathological manifestations found in the challenged group.

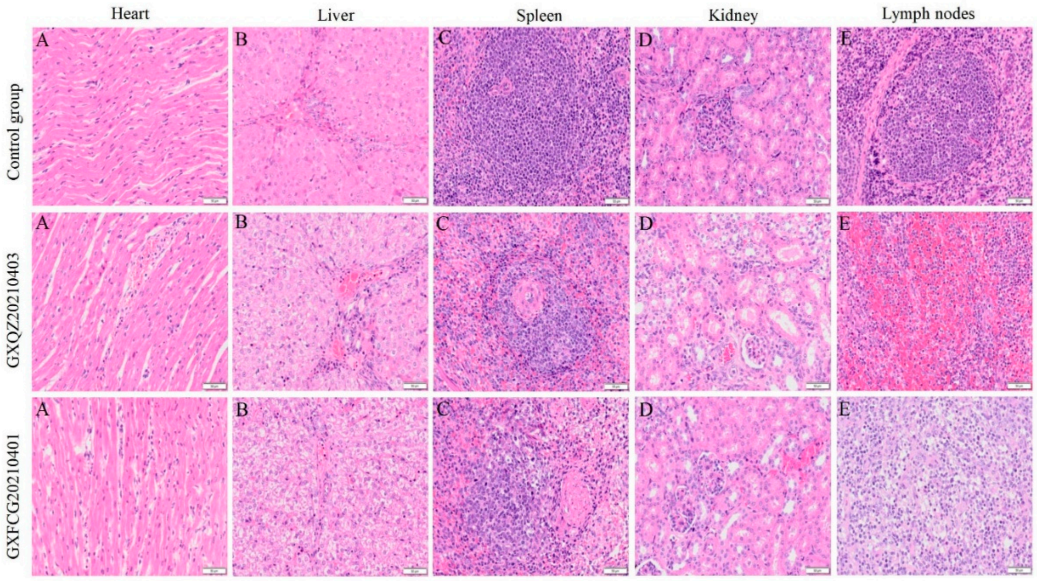

Figure S2. (**A**) Heart, a small amount of inflammatory infiltration was present in the myocardial tissues of the challenged groups when compared to the control group. (**B**) Liver, the tissues showed either obvious granular degeneration or severe vacuolar degeneration with inflammatory cell infiltration in the sub-lobular and portal areas in the challenged groups. (**C**) Spleen, vacuolar degeneration was noticed in the splenic trabeculae, marked haemorrhage in the red medullary area, blurred or even disintegrated splenic nodules, significant reduction in lymphocytes and accompanying

nuclear enrichment in the challenged groups. (**D**) Kidney, slight granular degeneration and haemorrhage were observed in the tubules, with lymphocytic infiltration in the tubular interstitium and mild damage to the basement membrane in the challenged groups. (**E**) Lymph nodes, haemorrhage was observed in some of the lymph nodes in the challenged groups. In addition, a large number of lymph nodes were structurally obscured or disintegrated, characterized by follicular and paracortical hyperplasia, particularly in the active proliferation of reticulocytes. The tissue slices were stained with hematoxylin eosin; Scale bar = 50 μm.

Table S1. Primers for amplification of the whole-length PRRSV genome.

Name of the Primer	Primer Sequence (5′-3′)	Position in Genome	Product Size(bp)
PRRSV-1-F	ATGACGTATAGGTGTTGGCTCT	1-1320	1320 bp
PRRSV-1-R	CCAGCACCATACCACTTATGA		
PRRSV-2-F	CTGGCAAGTACCTACAGCGGAG	1093-2219	1105 bp
PRRSV-2-R	CCAGGAAGCACAACATCCCAA		
PRRSV-3-F	CTAGGCTTGAGAAAGCTCGCC	2148-3610	1462 bp
PRRSV-3-R	ACAGGGAGATGGGAGACGA		
PRRSV-4-F	GATTCTGGAGGATCGCCGG	3456-4910	1454 bp
PRRSV-4-R	GCCAAGCCACCGATGGAAAC		
PRRSV-5-F	CACCAACCCGTTTGCCGT	4742-6153	1412 bp
PRRSV-5-R	GCTGCTGTTAGAAGCCTGATCATC		
PRRSV-6-F	GTGTGTGTTTTTCCTCCTGTGG	5954-7557	1604 bp
PRRSV-6-R	GGTTGTGCTCAACCGCGTC		
PRRSV-7-F	GTTGTTACTGAGACAGCGGT	7439-8970	1531 bp
PRRSV-7-R	ACCAGTGATAGTTTGGCATGG		
PRRSV-8-F	CTCCTGTTCCTACAGGATCAGC	8843-10348	1506 bp
PRRSV-8-R	AGTGTAACCACATCAAATGTGGC		
PRRSV-9-F	GAGGATGGCGCCATCACC	10289-11834	1546 bp
PRRSV-9-R	AAAGCATCCACAAAGAGTTGGCA		
PRRSV-10-F	TTTCGTGCGCGCCAGAAAG	11666-12945	1280 bp
PRRSV-10-R	GCCATTCTAGGTGAAACCAATTGCC		
PRRSV-11-F	GGTTGGCGTTCTTGTCCTT	12723-14367	1645 bp
PRRSV-11-R	CTGGAGGTGATGAATCTCCAGG		
PRRSV-12-F	GGGCTCTTGCACCTCTTAAT	14264-15110	847 bp
PRRSV-12-R	AATTTCGGCCGCATGGTTCTC		

Table S2. The detailed information of selected PRRSV reference strains.

Reference Strains	Country/Year	Accession No.	Reference Strains	Country/Year	Accession No.
Lelystad virus	EU/1991	M96262	FJWQ16	CHN/2016	KX758249
VR2332	USA/1995	AY150564	15HEB1	CHN/2015	KX815411
CH-1a	CHN/1996	AY032626	15ZJ1	CHN/2015	KX815432
CH-1R	CHN/2008	EU807840	SDyt1401	CHN/2014	MN642105
JXA1	CHN/2006	EF112445	SD-1602	CHN/2016	MH651743
JXA1 P80	CHN/2008	FJ548853	HNJYF-1606	CHN/2016	MH651738
CHSX1401	CHN/2014	KP861625	HBFL-1604	CHN/2016	MH651739
QYYZ	CHN/2010	JQ308798	HNJYH-1606	CHN/2016	MH651740
JA142	USA/2003	AY424271	JSWA	CHN/2014	KY373214
Ingelvac ATP	USA/2006	DQ988080	HENAN-XINX	CHN/2013	KF611905
DC	CHN/2010	JF748718	HENAN-HEB	CHN/2012	KJ143621
GX1002	CHN/2010	JQ955658	SCcd16	CHN/2016	MF196905
JL-04/12	CHN/2012	JX177644	SC-d	CHN/2015	MF375261
NT1	CHN/2014	KP179402	SD99-1606	CHN/2016	MH651745
TJ	CHN/2006	EU860248	XW015	USA/2013	KF724409
JXwn06	CHN/2008	EF641008	MN184A	USA/2005	DQ176019
JL580	CHN/2013	KR706343	MN184C	USA/2007	EF488739
Minnesota15	USA/2012	KP283405	SDSU60	USA/2014	KT258005
109560	USA/2014	KT257958	IA/2014/ISU-2	USA/2014	MF326989
HUN4	CHN/2007	EF635006	NC/2014/ISU-3	USA/2014	MF326990
SH1704-25	CHN/2017	MN119306	IA/2015/ISU-13	USA/2015	MF327000
NADC30	USA/2008	JN654459	NADC31	USA/2008	JN660150

WUH4	CHN/2011	JQ326271	OH155-2015	USA/2015	KR534894
WUH6	CHN/2011	KU523367	ISU51	USA/2014	KT257985
15HEN1	CHN /2015	KX815413	ISU17	USA/2014	KT257967
15LN1	CHN /2015	KX815423	IA/2014/NADC34	USA/2014	MF326985
HNhx	CHN /2016	KX766379	FJ0908	CHN/2018	MK202794
SDQD-1604	CHN /2016	MH651742	LNWK130	CHN/2017	MG913987
CY1-1604	CHN /2016	MH651736	LNWK96	CHN/2017	MG860516
CY2-1604	CHN/2016	MH651737	CH/2018/NCV-Anheal-1	CHN/2018	MH370474
PRR715664-S10-L001	USA/2019	MN073153	LNDZD10-1806	CHN/2018	MN648054
HeNLH2017	CHN/2017	MN823730	PRRSV-ZDXYL-China-2018-1	CHN/2018	MK453049
2035290	CAN/2017	MN865519	PRRSV-ZDXYL-China-2018-2	CHN/2018	MK453050
HB19-18	CHN/2019	MW651976	HLJZD22-1812	CHN/2018	MN648450
FJZ03	CHN/2015	KP860909	HLHDZD32-1901	CHN/2019	MN648449
HNyc15	CHN/2015	KT945018	HLJZD30-1902	CHN/2019	MN648055
HNjz15	CHN/2015	KT945017	RFLP 1-4-4 lineage 1C	USA/2020	MW887655
LNCH-1604	CHN/2016	MH651741	HENXX-1	CHN/2014	KU950372
FJY04	CHN/2015	KP860910	XW005	USA/2011	KF724398
FJ1402	CHN/2014	KX169191	ISU30	USA/2014	KT257977
HNhx 2016	CHN/2016	KX766379	46/2020	USA/2020	MZ423535
HENXC-4	CHN/2015	KU950371	PRRSV2-USA-Lab2	USA/2016	MT269878
SCnj16	CHN/2016	MF196906	15LN3	CHN/2015	KX815425
15SC3-1	CHN/2015	KX815428	HENXX-2014-12	CHN/2014	MN046228
SDbz16-2	CHN/2016	MH588710	HENXX-2014-3	CHN/2014	MN046226
HLJ/2017/921a	CHN/2017	MH422084	HENXX-2014-9	CHN/2014	MN046227
IA-2013-ISU-1	USA/2013	MF326988	SDYG1606	CHN/2016	KY053458
Minnesota1	USA/2012	KP283414	HEN1401	CHN/2014	MF766471
Peru/2015/18	PERU/2015	MH791380	15HEN4	CHN/2015	KX815415
OH-2014-ISU-6	USA/2014	MF326993	MN414	USA/2014	KT581982
IA/2015/ISU-9	USA/2015	MF326996	HNLCL82-1811	CHN/2018	MN648057
ISU29	USA/2014	KT257976	HNLCL58-1812	CHN/2018	MN648056
OH-2014-ISU-7	USA/2014	MF326994	NC/2015/ISU-11	USA/2015	MF326998
NCV-13	USA/2016	KX192112	ISU10	USA/2014	KT257966
ISU18	USA/2014	KT257968	NCV-25	USA/2016	KX192118
NC/2014/ISU-4	USA/2014	MF326991	NCV-26	USA/2016	KX192119
IA-2014-ISU-8	USA/2014	MF326995	HK4	CHN/2003	KF287134
IA/2015/NADC35	USA/2015	MF326986	SDZC-1609	CHN/2016	MH651747
IA/2015/NADC36	USA/2015	MF326987	GD-KP	CHN/2015	KU978619
IN-2014-ISU-5	USA/2014	MF326992	Neb-1	USA/2008	EU755263
IA/2015/ISU-10	USA/2015	MF326997	PrimePac	USA/1998	AF066384
NCV-21	USA/2016	KX192115	HK1	CHN/2003	KF287132
NCV-16	USA/2016	KX192113	HB-2(sh)-2002	CHN/2002	AY262352
NCV-17	USA/2016	KX192114	GM2	CHN/2011	JN662424
NCV-23	USA/2016	KX192116	PA8	CAN/2002	AF176348
NCV-24	USA/2016	KX192117	NADC-8	USA/1997	U66394

Table S3. Comparison of ORFs and amino acids of three PRRSV isolates with representative strains of other lineages or sub-lineages.

**Genome Region**	**Isolation of Strains**	**Reference of Strains（homology nt/aa%）**						
		**JXA1**	**CH-1a**	**VR2332**	**CHSX1401**	**NADC30**	NADC34	QYYZ
Complete genome	GXFCG20210401	85.1/	85.2/	85.0/	88.4/	88.7/	85.9/	83.0/
	GXQZ20210403	85.3/	85.1/	84.8/	88.6/	88.6/	86.0/	82.5/
	GXNN20210506	85.1/	85.1/	84.8/	88.1/	88.3/	85.8/	82.9/
5′UTR	GXFCG20210401	97.4	93.7	91.0	89.0	91.6	91.0	92.1
	GXQZ20210403	91.0	91.6	92.1	96.8	96.8	95.2	88.4
	GXNN20210506	96.8	93.2	91.0	89.5	91.1	91.5	91.6
NSP1α	GXFCG20210401	91.5/96.1	92.2/96.7	89.3/97.8	88.0/96.1	89.3/97.2	85.6/95.6	90.2/96.7
	GXQZ20210403	87.0/93.9	88.5/93.9	87.0/93.9	92.2/93.9	91.5/93.3	88.3/93.9	86.9/95.0
	GXNN20210506	90.9/95.6	91.7/96.1	89.1/97.2	87.4/95.6	88.7/96.7	85.0/95.0	90.0/96.1
NSP1β	GXFCG20210401	91.0/87.7	86.7/82.8	82.8/80.8	76.5/71.4	79.0/75.4	78.0/74.9	81.6/80.3
	GXQZ20210403	95.6/93.6	90.1/85.7	86.0/83.3	78.0/72.9	79.6/74.4	81.4/79.3	83.4/81.3
	GXNN20210506	91.6/88.7	87.4/83.7	82.9/81.8	76.5/71.9	79.1/75.4	78.3/74.9	82.3/81.3
NSP1	GXFCG20210401	91.2/91.6	89.4/89.3	85.7/88.8	81.8/83.0	83.9/85.6	81.5/84.6	85.6/88.0

	GXQZ20210403	91.6/93.7	89.3/89.6	86.5/88.3	84.782.8	85.2/83.3	84.7/86.2	85.0/87.7
	GXNN20210506	91.3/91.9	89.5/89.6	85.8/89.0	81.6/83.0	83.6/85.4	81.5/84.3	85.9/88.3
	GXFCG20210401	76.9/70.1	76.1/66.9	76.1/68.3	83.2/79.3	83.4/80.4	75.6/68.6	73.5/64.2
NSP2	GXQZ20210403	73.2/65.3	73.8/65.8	75.5/68.5	87.8/82.6	87.6/83.7	74.3/67.7	71.3/62.8
	GXNN20210506	77.0/70.0	76.1/66.5	76.0/68.0	83.1/79.0	83.4/80.1	75.7/68.7	73.5/64.2
	GXFCG20210401	81.7/90.4	82.9/90.8	83.6/91.3	91.0/96.2	90.2/95.5	83.9/91.3	80.9/87.4
NSP3	GXQZ20210403	84.4/91.3	84.5/92.2	84.8/91.5	88.9/94.4	89.5/94.4	84.5/91.3	80.1/87.9
	GXNN20210506	81.5/90.6	82.7/90.8	83.3/91.3	90.7/96.0	89.8/95.3	83.6/90.8	80.9/87.4
	GXFCG20210401	83.7/93.6	84.6/93.6	85.1/93.6	91.7/98.5	91.7/98.5	82.2/93.1	82.5/91.7
NSP4	GXQZ20210403	95.1/97.5	93.0/96.1	88.7/93.6	83.0/91.7	83.2/92.2	84.3/93.1	84.0/91.2
	GXNN20210506	83.7/93.6	85.0/93.6	85.1/93.6	91.5/98.5	91.3/98.5	82.2/93.1	82.2/91.7
	GXFCG20210401	85.9/93.5	88.6/91.8	86.3/91.8	90.4/90.6	90.4/92.4	82.2/88.8	82.0/88.8
NSP5	GXQZ20210403	92.7/92.9	90.4/90.6	86.1/88.8	86.7/91.2	84.9/88.8	82.0/88.2	81.2/90.0
	GXNN20210506	85.9/93.5	88.6/91.8	86.3/91.8	90.4/90.6	90.4/92.4	82.2/88.8	82.0/88.8
	GXFCG20210401	93.8/93.8	91.7/93.8	93.8/100	89.6/93.8	95.8/93.8	89.6/93.8	91.7/93.8
NSP6	GXQZ20210403	93.8/93.8	91.7/93.8	93.8/100	89.6/93.8	95.8/93.8	89.6/93.8	91.7/93.8
	GXNN20210506	93.8/93.8	91.7/93.8	93.8/100	89.6/93.8	95.8/93.8	89.6/93.8	91.7/93.8
	GXFCG20210401	79.4/82.6	80.7/83.8	83.5/86.5	90.3/91.9	90.5/92.3	82.1/86.5	78.8/82.6
NSP7	GXQZ20210403	94.5/94.2	91.5/91.9	86.6/86.5	81.3/81.1	80.6/81.1	80.8/84.9	90.7/91.9
	GXNN20210506	79.5/79.5	80.7/80.7	82.6/82.6	86.2/86.5	86.7/86.9	81.3/82.6	77.6/79.5
	GXFCG20210401	81.4/89.9	81.9/91.3	85.2/91.9	90.2/93.3	90.2/94.0	81.7/91.3	79.6/89.3
NSP7α	GXQZ20210403	93.3/93.3	92.2/93.3	87.7/91.9	83.0/87.9	82.8/87.2	83.0/90.6	89.3/91.3
	GXNN20210506	81.7/84.6	82.1/85.9	83.7/85.2	83.4/83.9	84.1/84.6	79.9/84.6	77.6/83.9
	GXFCG20210401	76.7/72.7	78.8/72.7	81.2/79.1	90.6/90.0	90.9/90.0	82.7/80.0	77.6/73.6
NSP7β	GXQZ20210403	96.1/95.5	91.5/90.9	85.2/79.1	79.1/71.8	77.6/72.7	77.9/77.3	92.7/92.7
	GXNN20210506	76.7/72.7	78.8/72.7	81.2/79.1	90.0/90.0	90.3/90.0	83.3/80.0	77.6/73.6
	GXFCG20210401	89.6/93.3	88.9/93.3	90.4/93.3	96.3/100	91.9/95.6	93.3/97.8	85.9/93.3
NSP8	GXQZ20210403	93.3/93.3	94.1/93.3	90.4/93.3	85.2/86.7	83.0/86.7	88.1/86.7	90.4/93.3
	GXNN20210506	89.6/93.3	88.9/93.3	90.4/93.3	96.3/100	91.9/95.6	93.3/97.8	85.9/93.3
	GXFCG20210401	92.0/97.5	90.5/97.2	89.1/97.3	89.8/96.2	89.2/96.2	88.2/97.3	88.4/96.7
NSP9	GXQZ20210403	86.8/96.7	86.7/95.6	86.4/95.8	91.7/97.2	91.1/96.6	87.4/95.6	85.1/94.8
	GXNN20210506	91.7/97.7	89.9/97.0	88.6/97.0	89.6/96.6	88.7/96.2	87.8/97.3	88.1/96.6
	GXFCG20210401	85.0/94.5	86.1/94.5	86.7/95.7	92.2/97.7	92.8/96.8	89.2/97.0	84.5/94.1
NSP10	GXQZ20210403	83.9/93.6	84.6/93.4	84.8/95.0	92.2/98.0	92.7/97.5	89.4/97.3	83.8/93.9
	GXNN20210506	84.8/93.9	85.8/93.9	86.2/95.0	91.7/97.7	92.1/96.8	88.9/96.6	84.2/93.4
	GXFCG20210401	89.3/95.5	91.1/96.9	88.7/95.1	89.0/94.2	91.7/96.9	86.9/95.1	87.2/95.1
NSP11	GXQZ20210403	88.2/96.0	90.9/96.4	88.2/94.2	88.8/93.8	92.6/96.4	87.1/94.2	86.6/93.8
	GXNN20210506	88.2/96.0	90.9/96.4	88.2/94.2	88.8/93.8	92.6/96.4	87.1/94.2	86.6/93.8
	GXFCG20210401	89.8/96.1	90.4/94.8	89.1/94.1	95.0/98.7	94.6/98.0	85.2/90.2	86.5/94.1
NSP12	GXQZ20210403	88.9/95.4	88.7/94.1	88.0/93.5	95.0/96.7	93.7/97.4	85.4/89.5	86.5/93.5
	GXNN20210506	90.2/95.4	90.8/94.1	89.3/93.5	94.6/98.0	94.1/97.4	85.0/89.5	86.1/93.5
	GXFCG20210401	86.3/83.7	87.4/86.0	87.5/84.4	85.1/85.6	85.3/85.2	96.0/93.8	85.7/83.3
ORF2a	GXQZ20210403	85.0/81.3	85.7/82.9	86.3/82.1	84.2/82.9	84.3/81.7	96.0/93.0	84.4/81.7
	GXNN20210506	86.3/84.0	87.4/86.4	87.5/84.8	85.1/86.0	85.3/85.6	95.7/93.4	85.7/83.7
	GXFCG20210401	87.4/85.1	87.8/85.1	88.3/86.5	89.6/90.5	90.5/91.9	96.8/98.6	87.4/90.5
ORF2b	GXQZ20210403	84.7/85.1	85.1/85.1	86.5/86.5	87.4/91.9	87.8/93.2	95.9/98.6	86.5/90.5
	GXNN20210506	87.8/85.1	88.3/85.1	88.7/86.5	90.1/90.5	91.0/91.9	96.4/98.6	87.8/90.5
	GXFCG20210401	82.5/80.8	82.6/80.0	82.6/81.6	84.7/82.4	83.7/82.7	93.7/94.5	82.1/82.0
ORF3	GXQZ20210403	83.3/82.4	83.4/81.6	84.2/82.7	84.8/82.7	84.1/83.1	94.5/94.9	83.0/83.1
	GXNN20210506	82.4/80.0	82.5/79.2	82.5/80.8	84.8/81.6	83.9/82.0	93.3/93.7	82.0/81.2
	GXFCG20210401	85.5/86.6	86.6/86.6	86.8/86.6	91.2/92.2	91.4/91.6	94.6/94.4	85.3/86.0
ORF4	GXQZ20210403	85.7/88.3	86.6/88.3	87.7/88.8	92.9/94.4	92.2/93.9	95.0/96.6	85.5/86.6
	GXNN20210506	85.8/88.3	87.0/88.3	87.2/88.3	92.4/95.0	92.4/94.4	95.2/97.2	85.3/86.6
	GXFCG20210401	86.1/87.1	86.9/86.6	86.6/85.1	86.9/89.1	86.7/89.0	96.0/95.5	83.6/82.1
ORF5	GXQZ20210403	85.9/88.6	86.7/88.1	86.4/86.6	87.2/90.0	86.7/90.0	96.7/96.5	83.6/83.1
	GXNN20210506	86.1/88.1	86.9/87.6	86.6/86.1	86.9/90.0	86.7/90.0	96.0/96.5	83.9/83.1
	GXFCG20210401	85.3/80.8	88.5/88.5	85.3/84.6	90.4/88.5	92.2/92.2	96.2/96.2	85.3/82.7
ORF5a	GXQZ20210403	85.3/75.0	88.5/82.7	86.5/80.8	91.7/86.5	92.2/86.3	97.4/94.2	86.5/80.8
	GXNN20210506	85.3/78.8	88.5/86.5	85.3/82.7	90.4/88.5	92.2/90.2	96.2/96.2	85.3/82.7
	GXFCG20210401	88.2/93.7	87.0/93.7	89.9/94.3	94.1/94.9	95.4/96.6	95.0/96.6	89.9/92.6
ORF6	GXQZ20210403	87.8/94.9	87.4/94.3	89.1/95.4	94.1/97.1	94.7/97.7	95.6/96.6	89.7/94.9
	GXNN20210506	87.8/93.1	86.7/93.1	89.5/93.7	93.5/94.3	94.9/96.0	94.5/96.0	89.5/92.0
ORF7	GXFCG20210401	88.4/88.7	89.5/91.1	90.9/92.7	92.7/90.3	92.7/93.5	94.6/94.4	89.0/91.1
	GXQZ20210403	90.3/91.1	90.3/92.7	91.9/93.5	94.9/92.7	96.0/97.6	94.9/93.5	87.6/90.3

	GXNN20210506	88.2/88.7	89.8/91.1	90.6/92.7	92.5/90.3	92.5/93.5	94.4/94.4	88.7/91.1
3′UTR	GXFCG20210401	88.7	87.4	90.7	96.0	97.3	94.7	89.4
	GXQZ20210403	88.7	86.8	90.1	95.4	96.6	94.0	87.4
	GXNN20210506	89.3	87.4	90.7	96.0	97.3	94.7	89.4

Table S4. Recombination events of PRRSV isolates GXQZ20210403，GXFCG20210401 and GXNN20210506 detected by RPD5 software.

Recombinant Strain	Breakpoints (Position in Alignment)		Major (Similarity)	Minor (Similarity)	p-Value of the Detection Methods						
	Beginning	Ending			RDP	GENECONV	BootScan	MaxChi	Chimaera	SiScan	3Seq
GXQZ20210403	660 (604-698)	1336 (1311-1365)	NADC30 (93.1%)	WUH4 (96.3%)	2.943×10^{-61}	1.073×10^{-43}	9.336×10^{-46}	2.544×10^{-17}	6.137×10^{-18}	2.515×10^{-17}	6.144×10^{-28}
	5302 (5251-5330)	8065 (8010-8152)	FJZ03 (91.0%)	JXA1-R (94.5%)	1.620×10^{-79}	1.703×10^{-31}	1.005×10^{-65}	2.422×10^{-27}	1.507×10^{-08}	5.271×10^{-30}	2.185×10^{-67}
	12092 (12050-12133)	14574 (14424-800)	FJWQ16 (90.9%)	HLJZD30-1902 (95.4%)	2.831×10^{-67}	6.788×10^{-35}	4.558×10^{-34}	2.307×10^{-23}	1.389×10^{-14}	1.304×10^{-28}	7.304×10^{-38}
	15422 (15337-56)	504 (475-528)	FJZ03 (91.6%)	SH1704-25 (97.6%)	2.606×10^{-19}	6.342×10^{-10}	1.182×10^{-15}	9.659×10^{-07}	3.576×10^{-08}	2.163×10^{-07}	NS
GXFCG20210401	696（652-754）	2044（1996-2048）	FJWQ16 (91.0%)	NT1 (84.6%)	5.624×10^{-60}	3.697×10^{-35}	1.202×10^{-55}	7.246×10^{-24}	8.569×10^{-11}	5.132×10^{-28}	3.194×10^{-35}
	7870 (7800-7891)	8786 (8751-8869)	FJZ03 (92.1%)	JL-04/12 (97.6%)	1.703×10^{-48}	7.701×10^{-43}	7.074×10^{-53}	7.939×10^{-15}	9.315×10^{-15}	5.047×10^{-18}	1.275×10^{-45}
	12088 (12044-12139)	14508 (14415-79)	FJWQ16 (89.3%)	HLJZD30-1902 (95.1%)	2.131×10^{-63}	2.200×10^{-28}	7.324×10^{-48}	2.088×10^{-22}	7.492×10^{-24}	9.051×10^{-26}	1.567×10^{-34}
	15433 (15337-56)	504 (475-528)	FJZ03 (89.5%)	SH1704-25 (97.0%)	2.606×10^{-19}	6.342×10^{-10}	1.182×10^{-15}	9.659×10^{-07}	3.576×10^{-08}	2.163×10^{-07}	NS
GXNN20210506	696（653-755）	2018（1996-2048）	FJWQ16 (91.0%)	NT1 (92.1%)	5.624×10^{-60}	3.697×10^{-35}	1.202×10^{-55}	7.246×10^{-24}	8.569×10^{-11}	5.132×10^{-28}	3.194×10^{-35}
	7851 (7800-7891)	8850 (8751-8869)	FJZ03 (91.5%)	JL-04/12 (96.7%)	1.703×10^{-48}	7.701×10^{-43}	7.074×10^{-53}	7.939×10^{-15}	9.315×10^{-15}	5.047×10^{-18}	1.275×10^{-45}
	12094 (12044-12139)	14507 (1415-79)	FJWQ16 (89%)	HLJZD30-1902 (95.0%)	2.131×10^{-63}	2.200×10^{-28}	7.324×10^{-48}	2.088×10^{-22}	7.492×10^{-24}	9.051×10^{-26}	1.567×10^{-34}

NS: not significant.
